# Cognitive Radar Waveform Optimization Based on Mutual Information and Kalman filtering

**DOI:** 10.3390/e20090653

**Published:** 2018-08-30

**Authors:** Yu Yao, Junhui Zhao, Lenan Wu

**Affiliations:** 1 School of Information Engineering, East China Jiaotong University, Nanchang 330031, China; 2 School of Information Science and Engineering, Southeast University, Nanjing 210096, China

**Keywords:** cognitive radar, target scattering coefficients (TSC), Kalman filtering, mutual information (MI), waveform optimization

## Abstract

A new strategy to optimizing the waveforms of cognitive radar under transmitted power constraint is presented. Our scheme is to enhance the performance of target estimation by minimizing the MSE (mean-square error) of the estimates of target scattering coefficients (TSC) based on Kalman filtering and then minimizing mutual information (MI) between the radar target echoes at successive time instants. The two steps are the optimal design of transmission waveform and the selection of a reasonable waveform from the ensemble for emission, respectively. The waveform design technique addresses the problems of target detection and parameter estimation in intelligent transportation system (ITS), where there is a need of extracting the features of target information obtained from different sensors. As the number of iterations increases, simulation results show better TSC estimation from the radar scene provided by the proposed approach as compared with the traditional waveform optimization algorithm. In addition, the proposed algorithm results in improved target detection probability.

## 1. Introduction

Cognitive radar (CR) can be considered as a perceptive system that would dynamically and intelligently shape its transmitted signals. It enables self-adaptively adjust its transmitted signal and filter based on all preceding information about radar targets and the surroundings, consequently has great potentials in improving its performance of target detection, estimation and recognition [1]. In recent years, radar theory has been linked with several new mathematical theories [2,3,4]. Crutchfield and Feldman developed an approach to applying information theoretic measures of randomness and memory to stochastic and deterministic processes by using successive derivatives of the Shannon entropy growth curve [5]. The prior knowledge comprises preceding measurements, assignment priorities and external databases. The target detection and estimation procedures provide an essential role in the environmental perception task, which are very important in intelligent waveform design [6]. Traditional radar systems would not take full advantage of the flexibility of system with the purpose of offering excellent performance of detection. In contrast, CR system has strong capacities in enhancing the target estimation and detection performance, which would show good performance in the face of various problems in a dynamic environment [7].

CR waveform optimization is an important means to enhance the performances of target estimation and detection. Bell [8] investigated the principle of information theory to optimize transmission waveform for detection of range spread target. A waveform optimization approach based on mutual information (MI) criterion is presented in the literature [9]. Mutual information (MI) criterion further improved the work of Bell. The work in [9] developed two kinds of waveform optimization criterions: MI minimization and mean-square error (MSE) minimization. The literature [10] focuses upon optimizing transmitted waveforms intended for MI minimization between the successive radar pulse echoes. In this way, the CR continually senses the environment and self-adaptively designs waveforms based on the MI minimization criterion.

Augusto and Antonio discussed the joint optimization problem of adaptive transmitting waveform and matched filter in various clutter environments [11,12]. Literature [13] presented a cognitive technique to optimize phase modulated waveforms sharing a desired range-Doppler response. The principle of the method is to minimize the mean value of the ambiguity function of the transmit waveform over a number of range Doppler bins. The approach intends to acquire more knowledge about target feature information. Considering range-ambiguous interference, the joint optimization problem of the transmitted waveform and matched filter for CR working in the complicated signal-dependent clutter environment was analyzed in [14]. The problem of transmitted code design was presented to improve the received signal to noise ratio (SNR) of range spread target [15]. In [16,17] a cognitive approach for transmitted waveform optimization based on the received SNR maximization was developed. Literature [18,19] presents a method of CR waveform optimization based upon the target detection probability maximization criterion, instead of the received SNR maximization. The similar methods are also proposed in [20,21,22,23]. Subject to a particular transmitting power constraint, literature [24,25] presents the water-filling method for allocating the transmitting power and maximizing the MI between target responses and measurements.

The parameters of transmitted signal are constantly adjusted with the intention of improving the performance of target estimation in the dynamic environment [26,27,28,29]. The similar researches on optimizing transmitted signals for CR systems include [30,31] which studied the equivalence between MI maximization stated in [24] and mean square error (MSE) of the target impulse response (TIR) minimization. Literature [32] presents a method of CR waveform optimization by maximizing the MI between the estimated TIR and the measurements. The approach would provide a better capability in detecting extended targets in the clutter environments. The radar target scattering characteristics can be considered as time-varying filter. Generally, the knowledge of radar environment is regarded as long-term memory, which is applied to CR waveform design over a relative long time. Such as, a waveform design scheme is proposed by minimizing the MI between the radar pulse echoes and the estimated TIR [33]. At the same time, a waveform design algorithm based on the MSE of the estimated TIR minimization criterion is proposed in [34]. Dai [35] proposes a Kalman filtering-based method, which intends to make use of the temporal correlation of target to obtain more target feature information. However, the method has low reliability and the computation is complex and not suitable for hardware implementation. Literatures [36,37,38,39,40] propose a waveform optimization approach by minimizing the target scattering coefficients (TSC) estimation to further enhancing the performance of detection. However, TSC varies constantly with the dynamic radar environment. From the point of self-adaptation system, the estimate of TSC should be constantly updated at the receiver, so that the system can learn faster and more accurately and adapt effectively to the changing environments. Literature [41,42] considers the waveform design method for the CR system. The TSC estimation based on the Kalman filtering is proposed, which can explore the time correlation of the TSC and gives a better estimation performance.

In this paper, we combine the temporal correlated algorithm presented in [41] and MI strategy [17] to obtain an optimization waveform, which offers superior radar performance. Specifically, we propose a new CR waveform design scheme. The proposed scheme is divided into two steps as follows.

Step 1: The step is to design an ensemble of optimization waveform for transmission. The main objective is to minimize the MSE of TSC subject to signal power and detection constraint [17]. The scheme ensures that the temporal correlation of the target feature information can be fully utilized at the receiver. Once the ensemble of optimization waveform is acquired, then we choose the most suitable waveforms for emission.

Step 2: The purpose of this module is to minimize the MI between the radar pulse echo at present and the next radar echo. This criterion ensures that we can acquire less dependent pulse echo signal samples to obtain more TSC information from pulse to pulse. Consequently, we choose the most suitable waveforms for transmission that produce uncorrelated and independent radar echoes. 

The key innovations of our work can be listed as follows:

(1) We present a Kalman filtering-based waveform design approach in order to take advantage of temporal correlation of TSC obtained from continual back-scattering echoes.
(2) We develop a new CR model, based on the idea of the MSE of TSC minimization.
(3) We propose a two-stage algorithm for waveform design in CR framework.
(4) We analyze the performances of the proposed CR system in regard to TSC estimation, target detection, receiver operating characteristics (ROC).

The organization of this paper is as follows. In Section 2, the system model of the proposed CR is formulated. In Section 3, TSC estimation based on maximum a posteriori (MAP) criterion is presented. In Section 4, a two-stage waveform optimization scheme is presented. The ensemble of transmission waveforms is designed in step 1 and the suitable waveform is selected from the ensemble in step 2. The simulation experiments demonstrating the two-stage waveform optimization schemes are presented in Section 5. The conclusion is given in Section 6.

Throughout our work, we use boldface lowercase letters and boldface uppercase letters to denote vectors and matrices, respectively; H to denote transpose conjugate operation; ${\left\|\right\|}_{2}$ to denote the $l_{2}$ norm; E{⋅} to denote the expectation operator; var{⋅} to denote the variance operator.

## 2. Cognitive Radar System Model

The transmitter sends wideband radar waveform to probe a range spread target and surroundings. The target echoes are gathered by the receive antenna and passed on to the matched filter, which matches the radar target echoes to transmitted waveform stored in the system receiver. The features of the backscattering information are examined for target parameter estimation. The Kalman filtering module designs an ensemble of optimization waveforms in the current instant and MI minimization module chooses an optimal signal from the assemble for the transmitter to obtain the best knowledge about the radar scene feature in the next radar pulse (presented in Section 4).

**Figure 1:**
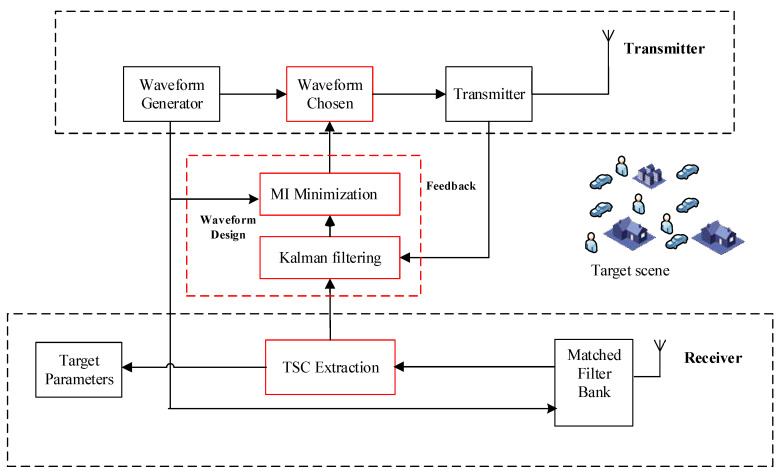
The architecture of the proposed CR system.

We discuss the performance of a CR system that includes the idea of “TCS estimation” and “MI Minimization.” The CR system proposed in this work is composed of four parts: Kalman filtering; MI minimization; Waveform chosen; TSC estimation. The parts of Kalman filtering and MI minimization are the new approach that distinguishes the differences between the proposed CR system and other types of feedback radar system. The architecture of the proposed CR system is stated in Figure 1.

We formulate CR system model in an intelligent transportation system (ITS) scenario, that is, the whole length of transmission signal is larger than the maximum delay relative to the first arrival from all the echoes, which is effective for short distance environmental perception applications when the distances of the transmitter via radar targets are within several hundred meters. We denote transmitted waveform that will be emitted as $f={\left[f\left(1\right),f\left(2\right),\ldots ,f\left(M\right)\right]}^{T}$, where M is the sample number. The total transmission energy is $\frac{1}{M}\sum_{m=1}^{M}{\left|f\left(m\right)\right|}^{2}=E_{f}$. Radar target is modeled as a range-extended target. The backscattering signal from the radar target is interfered by additive white Gaussian noise (AWGN) w. Therefore, the backscattering signal can be expressed in discrete form as

(1) y=Zg+w

where Z=diag{z} describes a diagonal matrix of transmitted waveform in the frequency domain. z denotes the Fourier transform of transmitted waveform f. g is the TSC vector, which is the Fourier transform of TIR vector. w is the additive Gaussian noise in the frequency domain. We define $E\left[g^{H}g\right]=R_{T}$ and $E\left[ww^{H}\right]=R_{N}$ as the covariance matrices of TSC and AWGN. We have y~N{0,R}, where

(2) $$\begin{array}{cc} R & =E\left\{yy^{H}\right\}\\ & \;=E\left\{Zgg^{H}Z^{H}+ww^{H}\right\}\\ & \;=ZR_{T}Z^{H}+R_{N} \end{array}$$

If the target does not exist, we have $y\sim N\left\{0,R_{N}\right\}$. From the literature [35], the time dynamic characteristic of the TSC at time i can be described as

(3) $$g_{i}=e^{-T/\tau }g_{i-1}+v$$

where i denotes the index of radar pulses. τ is the time correlation coefficient of TSC during pulse recurrence interval. T denotes the pulses interval. v describes complex Gaussian noise with zero-mean.

## 3. TSC Estimation

Target feature estimation can be considered as the prerequisite condition for the target detection. We intend to estimate TSC for enhancing the detection performance of CR system. We assume TSC is random and follows a Gaussian distribution. Generally, the maximum a posterior (MAP) criterion is used to estimate the TSC. The TSC estimation algorithm at time i is expressed as

(4) $$\begin{array}{cc} {\hat{g}}_{i} & =\arg\underset{g_{i}}{\max}p\left(g_{i}|y_{i}\right)\\ & =\arg\underset{g_{i}}{\max}\frac{p\left(y_{i}|g_{i}\right)p\left(g_{i}\right)}{p\left(y_{i}\right)} \end{array}$$

where

(5) $$\begin{array}{c} p\left(y_{i}|g_{i}\right)=\frac{1}{{\left(2\pi \right)}^{M/2}{\left|R_{N}\right|}^{1/2}}\exp\left(-\frac{1}{2}{\left(y_{i}-Z_{i}g_{i}\right)}^{H}R_{{}_{N}}^{-1}\left(y_{i}-Z_{i}g_{i}\right)\right)\\ p\left(g_{i}\right)=\frac{1}{{\left(2\pi \right)}^{M/2}{\left|R_{T}\right|}^{1/2}}\exp\left(-\frac{1}{2}{\left(g_{i}\right)}^{H}R_{{}_{T}}^{-1}g_{i}\right)\\ p\left(y_{i}\right)=\int p\left(y_{i}|g_{i}\right)p\left(g_{i}\right)dg_{i} \end{array}$$

By substituting (5) into (4), the posterior probability $p\left(g_{i}|y_{i}\right)$ can be obtained as follows:

(6) $$\begin{array}{c} p\left(g_{i}|y_{i}\right)=\frac{p\left(y_{i}|g_{i}\right)p\left(g_{i}\right)}{p\left(y_{i}\right)}\\ =\frac{\exp\left({\left(Z_{i}g_{i}\right)}^{T}y_{i}-\frac{1}{2}{\left(Z_{i}g_{i}\right)}^{T}Z_{i}g_{i}-\frac{1}{2}{\left(g_{i}\right)}^{T}R_{T}^{-1}\left(g_{i}\right)\right)}{\exp\left(\frac{1}{2}{\left(\frac{{\left(Z_{i}\right)}^{T}y_{i}}{R_{{}_{N}}^{}}\right)}^{T}{\left({\left(Z_{i}\right)}^{T}Z_{i}+R_{T}^{-1}\right)}^{-T}\left(\frac{{\left(Z_{i}\right)}^{T}y_{i}}{R_{{}_{N}}^{}}\right)\right)\sqrt{\left|2\pi {\left({\left(Z_{i}\right)}^{T}Z_{i}+R_{T}^{-1}\right)}^{-1}\right|}} \end{array}$$

The backscattering signals $y_{i}$ follows Gaussian distribution and the posterior probability $p\left(g_{i}|y_{i}\right)$ denotes probability distribution function of TSC. Thus, the estimate of TSC can be written as follows:

(7) $$\begin{array}{cc} {\hat{g}}_{i} & =\underset{g_{i}}{\arg\max}\left\{g_{i}^{H}\left(Z_{i}^{H}R_{N}^{-1}Z_{i}+R_{T}^{-1}\right)g_{i}-y_{i}^{H}R_{N}^{-1}Z_{i}g_{i}-g_{i}^{H}Z_{i}^{H}R_{N}^{-1}y_{i}\right\}\\ & \;=C_{i}y_{i} \end{array}$$

The receiver filter can be denoted by the matrix form $C_{i}$ as follows

(8) $$C_{i}={\left[{\left({Ζ}_{i}\right)}^{H}C_{N}^{-1}{Ζ}_{i}+C_{T}^{-1}\right]}^{-1}{\left({Ζ}_{i}\right)}^{H}C_{N}^{-1}$$

The MSE of TSC estimation can be written as follows:

(9) $$\begin{array}{cc} P_{i} & =E\left\{{\left\|{\hat{g}}_{i}-g_{i}\right\|}_{2}^{2}\right\}\\ & \;=E\left\{\left(C_{i}y_{i}-g_{i}\right){\left(C_{i}y_{i}-g_{i}\right)}^{H}\right\}\\ & \;=C_{i}\left(Z_{i}R_{T}{\left(Z_{i}\right)}^{H}+R_{N}\right){\left(C_{i}\right)}^{H}-C_{i}Z_{i}R_{T}-R_{T}{\left(Z_{i}\right)}^{H}{\left(C_{i}\right)}^{H}+R_{T} \end{array}$$

## 4. Waveform Optimization

We develop a new scheme to optimizing the CR waveform. The first step: The primary purpose of waveform optimization is to minimize the estimate of TSC via Kalman filtering. Once an ensemble of optimized signals is acquired, then we intend to choose the most suitable waveform for probing from the optimized ensemble. The second step: We develop a waveform selection criterion on the basis of minimizing the MI between the radar pulse echo at present and the next radar echo. The successive backscattering signals are statistically independent on each other in time, in order to obtain more radar scene feature information at each radar pulse.

### 4.1. TSC Estimation Based on Kalman Filtering

Generally, the TSC is considered as time variant and transmitted waveform should be adaptively adjusted accordingly. In order to make good use of the temporal sequence correlation of target feature during the pulses interval, we develop a scheme for TSC estimation based on Kalman filtering in the first step. The iterative procedure of Kalman filtering based TSC estimation can be summarized as Algorithm 1.

**Algorithm 1.** The MSE of TSC estimation matrix based on Kalman filtering **Step 1:** Initialize the MSE of TSC $P_{1/1}=0$
**Step 2:** At time i−1, calculate the predicted TSC as follows: ${\hat{g}}_{i|i-1}=e^{-T/\tau }{\hat{g}}_{i-1|i-1}$ **Step 3:** At time i−1, calculate the predicted MSE of TSC as follows: $P_{i|i-1}=e^{-2T/\tau }P_{i-1|i-1}+\left(1-e^{-2T/\tau }\right)R_{T}$ **Step 4:** At time i, calculate the Kalman gain as follows: $\Phi_{i}=P_{i|i-1}{\left(C_{i}Z_{i}\right)}^{H}{\left(C_{i}R_{N}C_{i}{}^{H}+C_{i}Z_{i}P_{i|i-1}Z_{i}{}^{H}C_{i}{}^{H}\right)}^{-1}$ **Step 5:** At time i, calculate the estimated TSC as follows: ${\hat{g}}_{i|i}={\hat{g}}_{i|i-1}+\Phi_{i}\left({\hat{g}}_{i}-C_{i}Z_{i}{\hat{g}}_{i|i-1}\right)$ **Step 6:** At time i, update the MSE of TSC as follows: $P_{i|i}=P_{i|i-1}-\Phi_{i}C_{i}Z_{i}P_{i|i-1}$ If $i=I_{\max}$, the iterative procedure ends; or else, return to Step 2 and repeat.

We optimize transmitted waveform by minimizing the MSE matrix of TSC at each Kalman filtering step. The CR waveform optimization problem is preliminary presented as follows:

(10) $$\begin{array}{c} z_{i}=\underset{z_{i}}{\arg\min}\left\{tr\left(P_{i|i}\right)\right\}\\ s.t.\;z_{i}^{H}z_{i}\leq E_{f}\\ \;P_{D}\geq \varepsilon \end{array}$$

The objective function in (10) is to minimize the MSE of TSC matrix and the constraint function in (10) is the probability of target detection and transmitting power. We simplify the probability of target detection constraint. The likelihood ratio detection can be expressed as:

(11) $$l\left(y_{i}\right)=y_{i}^{H}R_{N}^{-1}Z_{i}{\hat{g}}_{i}\overset{H_{1}}{\underset{H_{2}}{≶}}T$$

where T is the detection threshold, which is assumed to be a constant. Hence, by substituting (1) into (11), the probability of target detection can be obtained as follows:

(12) $$\begin{array}{l} P_{d}=P\left({\left(Z_{i}{\hat{g}}_{i}+v\right)}^{H}R_{N}^{-1}\left(Z_{i}{\hat{g}}_{i}\right)\geq T\right)\\ =Q\left(Q^{-1}\left(P_{fa}\right)-{\left(Z_{i}{\hat{g}}_{i}\right)}^{H}R_{N}^{-1}Z_{i}{\hat{g}}_{i}\right) \end{array}$$

where $P_{fa}=Q\left(\frac{T}{\sqrt{{\left(Z_{i}{\hat{g}}_{i}\right)}^{H}R_{N}^{-1}Z_{i}{\hat{g}}_{i}}}\right)$ is the false alarm probability. Q(⋅) denotes the *Q*-function. Because Q(x) is a monotonic decreasing function of x, we can re-express the detection probability as follows:

(13) $$p\left(z_{i}\right)=z_{i}^{H}{\hat{G}}_{i}{}^{H}R_{N}^{-1}{\hat{G}}_{i}z_{i}\geq \varepsilon^{\prime }$$

where ${\hat{G}}_{i}=diag\{{\hat{g}}_{i}\}$. According to the Algorithm 1, we can re-express the objective function as follows:

(14) $$f\left(z_{i}\right)=tr\left({\left({\left(P_{i|i-1}\right)}^{-1}+Z_{i}^{H}R_{N}^{-1}Z_{i}\right)}^{-1}\right)$$

Therefore, we can re-express CR waveform design problem (10) as follows:

(15) $$\begin{array}{l} z_{i}=\arg\underset{z_{i}}{\min}\left\{tr\left({\left({\left(P_{i|i-1}\right)}^{-1}+Z_{i}^{H}R_{N}^{-1}Z_{i}\right)}^{-1}\right)\right\}\\ s.t.\;z_{i}^{H}z_{i}\leq E_{f}\\ \;z_{i}^{H}{\hat{G}}_{i}{}^{H}R_{N}^{-1}{\hat{G}}_{i}z_{i}\geq \varepsilon^{\prime } \end{array}$$

where $p\left(z_{i}\right)=z_{i}^{H}{\hat{G}}_{i}{}^{H}R_{N}^{-1}{\hat{G}}_{i}z_{i}$ is the target detection constraint. By performing Eigen-decomposition (ED) to $p\left(z_{i}\right)=z_{i}^{H}{\hat{G}}_{i}{}^{H}R_{N}^{-1}{\hat{G}}_{i}z_{i}$, eigenvalues’ distribution in signal subspace can be obtained [27]. While $z_{i}$ denotes the eigenvector of ${\hat{G}}_{i}{}^{H}R_{N}^{-1}{\hat{G}}_{i}$ with the maximum eigenvalue, we have $\max p\left(z\right)=\lambda_{\max}E_{f}$, where $\lambda_{\max}$ is the maximum eigenvalue of ${\hat{G}}_{i}{}^{H}R_{N}^{-1}{\hat{G}}_{i}$, $E_{f}=v_{\max}^{H}v_{\max}$, $v_{\max}$ is the corresponding maximum eigenvector. Therefore, the CR waveform design problem (10) is convex satisfying the condition $\lambda_{\max}v_{\max}^{H}v_{\max}\geq \varepsilon^{\prime }$. We further assume that $P_{i|i-1}$ and $R_{N}$ are diagonal matrix. We have

(16) $$\begin{array}{l} z_{i}=\arg\underset{z_{i}}{\min}\sum_{m}\frac{P_{i|i-1,m}R_{N,m}}{R_{N,m}+z_{i,m}^{H}z_{i,m}P_{i|i-1,m}}\\ s.t.\;z_{i}^{H}z_{i}\leq E_{f}\\ \;z_{i}^{H}{\hat{G}}_{i}{}^{H}R_{N}^{-1}{\hat{G}}_{i}z_{i}\geq \varepsilon^{\prime } \end{array}$$

where $z_{i,m}$ denotes the *m*-th entry of $z_{i}$, $P_{i|i-1,m}$ and $R_{N,m}$ denote the *m*-th row and *m*-th column of $P_{i|i-1}$ and $R_{N}$, respectively. To solve the optimization problem in (16), we use the Lagrange function as

(17) $$f\left(z_{i}^{},\lambda_{1},\lambda_{2}\right)=\sum_{m}\frac{P_{i|i-1,m}R_{N,m}}{R_{N,m}+z_{i,m}^{H}z_{i,m}P_{i|i-1,m}}+\lambda_{1}\left(p\left(z_{i}\right)-\varepsilon^{\prime }\right)+\lambda_{2}\left(z_{i}^{H}z_{i}-E_{f}\right)$$

where $p\left(z_{i}\right)=z_{i}^{H}{\hat{G}}_{i}{}^{H}R_{N}^{-1}{\hat{G}}_{i}z_{i}$. The extreme value of (17) can be attained using

(18) $$\begin{array}{l} \frac{\partial f\left(z_{i},\lambda_{1},\lambda_{2}\right)}{\partial {\left\|z_{m}\right\|}^{2}}=0\\ \Rightarrow \frac{P_{i|i-1,m}R_{N,m}P_{i|i-1,m}}{{\left(R_{N,m}+z_{i,m}^{H}z_{i,m}P_{i|i-1,m}\right)}^{2}}+\lambda_{1}\left({\hat{G}}_{i}{}^{H}R_{N}^{-1}{\hat{G}}_{i}\right)+\lambda_{2}=0 \end{array}$$

With $L_{i,m,m}$ denoting the entry at *m*-th row and *m*-th column of $L_{i}$. Hence, the optimization waveform can be obtained as follows

(19) $${\left\|z_{m}\right\|}_{2}^{2}=\max\left\{\sqrt{\frac{R_{N,m}}{\lambda_{1}\left({\hat{G}}_{i}{}^{H}R_{N}^{-1}{\hat{G}}_{i}\right)+\lambda_{2}}}-\frac{R_{N,m}}{P_{i|i-1,m}},0\right\}$$

where $\lambda_{1}$ and $\lambda_{2}$ are utilized to restrain the power of transmitted waveform and the detection probability.

### 4.2. MI Minimization between the Radar Pulse Echo at Present and the Next Radar Pulse Echo

We denote the MI between the radar pulse echo at present $y_{i}$ and the next radar pulse echo $y_{i+1}$ as $I\left(y_{i},y_{i+1}\right)$. If $y_{i}$ and $y_{i+1}$ are statistically dependent, $I\left(y_{i},y_{i+1}\right)$ would be extremely high. Thus, high gain in target feature information cannot be obtained. We proceed to the waveform selection module, in which we mean to acquire independent and uncorrelated radar pulse samples reflected by the target to gain more TSC information from scan to scan. Subsequently, we choose the most suitable waveforms for emission that produce less statistically dependent backscattering signals. In other words, we desire to obtain the reasonable waveform from the ensemble that would minimize $I\left(y_{i},y_{i+1}\right)$.

We denote $y_{i}={\left[y_{i}\left(1\right),y_{i}\left(2\right),\ldots ,y_{i}\left(M\right)\right]}^{T}$ as the vector of backscattering signal at time i. $I\left(y_{i},y_{i+1}\right)$ can be expressed as

(20) $$I\left(y_{i},y_{i+1}\right)=I\left(y_{i}|z_{i}\right)+I\left(y_{i+1}|z_{i+1}\right)-I\left(y_{i},y_{i+1}|z_{i},z_{i+1}\right)$$

where $H\left(y_{i}|z_{i}\right)$ denotes the entropy of $y_{i}$ given the knowledge of the transmitted waveform $z_{i}$. The terms $H\left(y_{i+1}|z_{i+1}\right)$ and $H\left(y_{i},y_{i+1}|z_{i},z_{i+1}\right)$ in (20) are defined similarly.

We denote $y=\left\{y_{i}\left(m\right),y_{i+1}\left(m\right)\right\}$ as the *m*-th sample sequence of the vector of two successive received signals. The probability density function of y can be expressed as

(21) $$f_{y}\left(y\right)=\frac{1}{{\left(\sqrt{2\pi }\right)}^{2}{\left|\Sigma \right|}^{\frac{1}{2}}}\exp\left[\frac{{\left(y-\mu \right)}^{H}{\left|\Sigma \right|}^{-1}\left(y-\mu \right)}{2}\right]$$

H(y|z) is presented as follows:

(22) $$I\left(y|z\right)=1+\frac{1}{2}In\left[{\left(2\pi e\right)}^{2}\left|\Sigma \right|\right]$$

Hence, $H\left(y_{i},y_{i+1}|z_{i},z_{i+1}\right)$ can be derived as follows

(23) $$\begin{array}{cc} I\left(y_{i},y_{i+1}|z_{i},z_{i+1}\right) & =I\left(y_{i}\left(m\right),y_{i+1}\left(m\right)|z_{i}\left(m\right),z_{i+1}\left(m\right)\right)\\ & =1+\frac{1}{2}In\left[{\left(2\pi e\right)}^{2}\left|\Sigma \right|\right] \end{array}$$

The covariance matrix can be denoted as

(24) $$\Sigma =\left|\begin{array}{cc} R_{i,}^{2} & \rho_{i,i+1}R_{i,}R_{i+1,}\\ \rho_{i,i+1}R_{i,}R_{i+1,} & R_{i+1,}^{2} \end{array}\right|$$

where $\left|\Sigma \right|=R_{i}^{2}R_{i+1}^{2}\left(1-\rho_{i,i+1}^{2}\right)$, and $\rho_{i,i+1}=\frac{E\left[y_{i}^{H}y_{i+1}\right]}{\sqrt{\delta_{i}^{2}\delta_{i+1}^{2}}}$ is the correlation coefficient. The term $H\left(y_{i}|z_{i}\right)$ can be derived as follows

(25) $$\begin{array}{cc} I\left(y_{i}|z_{i}\right) & =-\int \Psi \left(y\right)In\left[\Psi \left(y\right)\right]dy\\ & =-\int \Psi \left(y\right)In\left[-\frac{{\left(y-\mu_{i}\right)}^{2}}{2R_{i}^{2}}-In\left(\sqrt{2\pi R_{i}^{2}}\right)\right]dy\\ & =\frac{1}{2}+\frac{1}{2}In\left(2\pi R_{i}^{2}\right) \end{array}$$

where $\Psi \left(y\right)=\frac{1}{\sqrt{2\pi R_{i}^{2}}}\exp\left[-\frac{{\left(y-\mu_{i}\right)}^{2}}{2R_{i}^{2}}\right]$ is the univariate pdf of $y_{i}$. Similarly, the term $H\left(y_{i+1}|z_{i+1}\right)$ can be defined as follows:

(26) $$I\left(y_{i+1}|z_{i+1}\right)=\frac{1}{2}+\frac{1}{2}In\left(2\pi R_{i+1}^{2}\right)$$

Then, we can obtain $I\left(y_{i},y_{i+1}\right)$ by substituting (23), (25), (26) into (20)

(27) $$\begin{array}{cc} I\left(y_{i},y_{i+1}\right) & =\frac{1}{2}In\left(2\pi R_{i}^{2}\right)+\frac{1}{2}In\left(2\pi R_{i+1}^{2}\right)-\frac{1}{2}In\left({\left(2\pi \right)}^{2}R_{i}^{2}R_{i+1}^{2}\left(1-\rho_{i,i+1}^{2}\right)\right)\\ & =-\frac{1}{2}In\left(1-\rho_{i,i+1}^{2}\right) \end{array}$$

Finally, the minimization MI problem can be formed as follows

(28) $$\begin{array}{c} I_{\min}=\underset{z_{i+1}\in C_{z_{{}_{i}}}}{\min}\left\{-\frac{1}{2}In\left(1-\rho_{i,i+1}^{2}\right)\right\}\\ s.t.\;\mathrm{tr}\left[z_{i+1}^{H}z_{i+1}\right]\leq E_{f} \end{array}$$

The backscattering signal $y_{i}$ is exploited to estimate the covariance matric of the TSC $R_{T}$. The received signal $y_{i+1}$ over all possible transmitted waveform $z_{i+1}\in C_{z_{i}}$ can be estimated by using (1). We generate an estimate of all the received signals of the corresponding $\rho_{i,i+1}$. Therefore, the minimization MI problem (28) can be solved by choosing the value for $z_{i+1}\in C_{z_{i}}$.

The first step: we design optimization waveform for transmission with an objective of minimizing the MSE of TSC estimation via Kalman filtering; the second step: We choose the most suitable waveform based on MI minimization between successive target echoes. The proposed waveform optimization process is presented as Algorithm 2.

**Algorithm 2.** The proposed CR waveform design algorithm**Step 1:** Initialize the observed state of TSC $g_{1}$ and the MSE of TSC $P_{1/1}=0$. **Step 2:** Solve for the optimization waveforms ensemble for transmission with an objective of minimizing the MSE of TSC as stated in step one. **Step 3:** Generate the estimated value $y_{2}$ based on the estimated value ${\hat{g}}_{2}$. **Step 4:** Solve for optimization waveform $z_{2}$ on the basis of the MI minimization criterion as stated in step two. **Step 5:** Update the observed state of TSC $g_{2}$ based on the current value $y_{2}$. **Step 6:** If $i=I_{\max}$, the iterative procedure ends; or else, return to 2 and repeat.

We can summarize the two steps cognitive waveform design scheme as follows:

(1) The proposed CR continuous updates the estimate of TSC by continual measurements of TSC and employs the radar scene feature to select the most suitable signal for emission. The cognitive receiver is able to the delivery of the TSC estimation to the transmitter.
(2) The transmitter adaptively adjusts its probing signal to accommodate the unknown dynamic environment.

**Figure 2:**
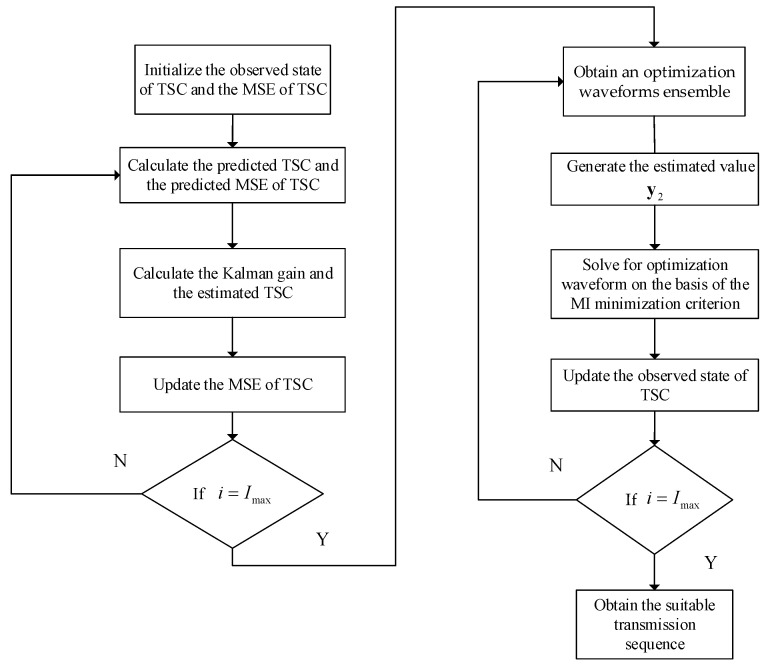
The two steps optimization scheme for target estimation.

In summary, the proposed two steps optimization scheme for target estimation can be implemented according to the block diagram in Figure 2.

## 5. Simulation

The process of the two steps waveform design scheme is shown in Figure 1. The performances improvement of target detection and TSC estimation provided by the proposed algorithm are discussed in this section. For a dynamic target scene, 900 simulations wave always run for each test at particular received SNRs. The experiment parameters are presented in Table 1.

The TSC is estimated via Kalman filtering in the subsequent time interval. The normalized MSE of TSC is used for defining the performance of target estimation as follows:

(29) $$nMSE=\frac{{\left\|\hat{g}-g\right\|}_{2}^{2}}{{\left\|g\right\|}_{2}^{2}}$$

where $\hat{g}$ and g denote the estimate for TSC and the observed state of TSC, respectively. 

**Figure 3:**
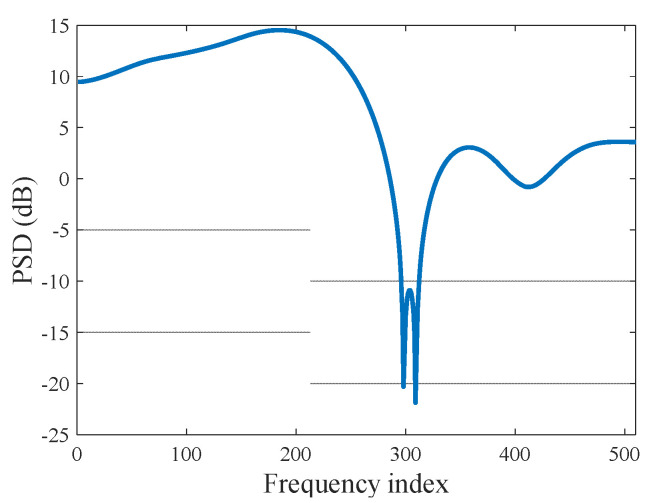
The PSD of the target.

We assume that the length of the target impulse response is N=32 range cells. The PSD of the target is shown in Figure 3. In our simulations, the AWGN is given. The initial range of the target is $R_{0}=1\;\mathrm{km}$ and the velocity is v=60 km/h. The target deviates from the radar along the line of sight (LOS) and the initial SNR is 15 dB.

### 5.1. Target Detection Performance

**Figure 4:**
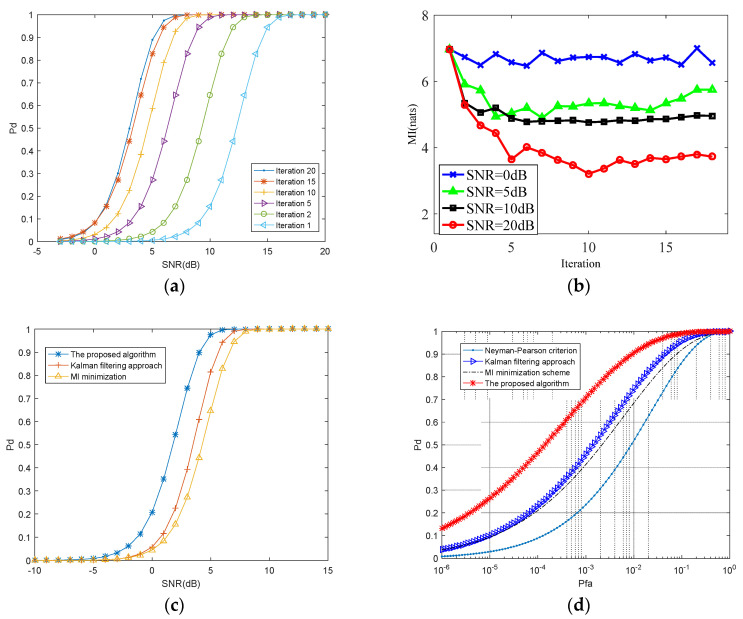
(**a**) The probability of target detection for different iterations of Kalman filtering scheme; (**b**) The probability of target detection for different SNRs of MI minimization scheme; (**c**) The probability of target detection for 3 kinds of schemes; (**d**) The ROC for 4 kinds of strategies.

Figure 4a demonstrates the probability of target detection provided by the Kalman filtering approach for different SNRs. The optimization waveform is generated by Kalman filtering approach. The iterative procedure has been run for 20 times. As shown in Figure 4a, the value of required SNR decreases as the iteration times increase for a particular target detection probability. The Kalman filtering approach converges after 15 iterative computations, yielding a probability of 0.9 at SNR = 3 dB as compared to SNR = 13 dB at the beginning of the iteration. The detection capability of Kalman filtering approach is becoming better as the number of iterations increases. Nonetheless, target detection probability does not present more enhancements after 20 iterative computations.

Figure 4b shows the detection probability provided by the MI minimization scheme in different values of SNR environment. The covariance matrix of TSC $R_{T}$ and the correlation coefficient $\rho_{i,i+1}$ can precisely be estimated at high SNRs. As a consequence, the most suitable transmitted signal for the next radar pulse can be exactly selected. Hence, the value of MI reduces as the number of iterations rises. In contrast, the estimate for $R_{T}$ and $\rho_{i,i+1}$ are poor at low SNRs. The MI value does not present obviously reduce with many iterative computations. Therefore, there is little improvement in the target estimation with regard to the dynamic radar environment. MI minimization scheme cannot offer performance enhancement in this case. 

In Figure 4c, we compare detection probability for the optimized waveform offered by the two steps algorithm with detection probability for the optimized waveform provided by MI minimization scheme and compare the result with Kalman filtering approach as well. The iterative procedure has been run for 20 times. Since the proposed algorithm utilizes the Kalman filtering and MI minimization from pulse to pulse, CR system dynamically adapts radar waveforms is superior to waveform provided by MI minimization scheme. Besides, Kalman filtering approach cannot acquire uncorrelated radar pulse samples reflected by the target to gain more TSC information after 20 iterative computations. As you can see in Figure 4c, detection probability of Kalman filtering approach and MI minimization scheme are suboptimal and detection probability of the proposed two steps algorithm (combining Kalman filtering algorithm and MI strategy) is optimal.

Figure 4d demonstrates the ROC for 4 types of strategies. (1) Traditional radar system based on MAP criterion; (2) CR system based on MI minimization criterion as stated in [27]; (3) CR system based on Kalman filtering as stated in [42]; (4) CR system using the proposed waveform design algorithm.

The curves for the Kalman filtering approach, the MI minimization scheme and the two steps algorithm have been operated at the end of 25 iterative computations. For $P_{fa}=0.01$, the probability of detection provided by the two steps algorithm is 0.9 as compared with 0.7 generated by MI minimization scheme, 0.65 by Kalman filtering approach and 0.5 by MAP criterion. As the proposed algorithm utilize the temporal correlation of TSC during the pulses interval, the proposed radar system adapts its probing signal to the fluctuating target RCS. Besides, the successive scattered signals are uncorrelated with on each other. This ensures that something more about the target information can be learned at every instant of reception. Therefore, the detection probability of the proposed two steps algorithm (combining Kalman filtering algorithm and MI strategy) is optimal.

### 5.2. TSC Estimation Performance

**Figure 5:**
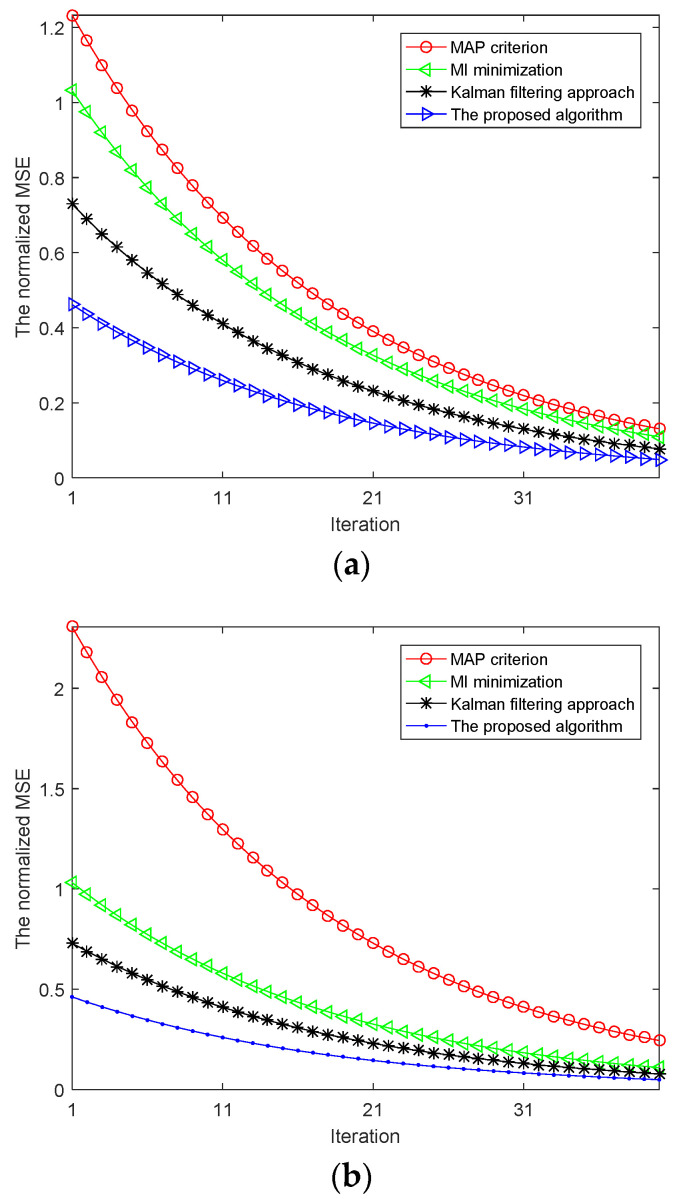
(**a**) The MSE of TSC estimation subject to signal power constraint; (**b**) The MSE of TSC estimation subject to signal power and probability constraint.

Figure 5a shows the normalized MSE provided by the algorithm with respect to TSC estimation subject to the signal power constraint. The curves indicate an enhanced estimation performance for the two steps algorithm as compared with the MAP criterion, Kalman filtering approach and MI minimization scheme. As shown in the Figure 5a, the MSE performance of TSC provided by the two steps algorithm is better than MAP criterion and Kalman filtering approach. This can be fulfilled by choosing transmitted waveforms that result in uncorrelated and independent radar pulse samples in order to obtain more target feature information. At the same time, the MSE performance of TSC provided by the two steps algorithm is better than MI minimization scheme at each iteration step. 

Figure 5b demonstrates the normalized MSE provided by the algorithm with respect to TSC estimation subject to the signal power and detection probability constraint. The MSE of TSC achieved by the proposed algorithm and three different schemes are compared to demonstrate the advantage of the two steps algorithm, particularly for the first few iterations.

## 6. Conclusions

We have developed a CR waveform optimization algorithm, which includes the waveform optimization module and waveform selection module. The two steps scheme is based upon continuous perceiving of the target and surroundings via Kalman filtering and adaptation of the send signals to suit the time-variant surroundings. Such a cognitive strategy ensures better target discrimination and parameter estimation capability. The simulation results demonstrate that, the proposed CR waveform design scheme provides great performance improvements in regard to detection probability and TSC estimation. Such cognitive radar system can be used in ITS applications to address the environmental sensing issues. However, there is a raise in the operational cost because of the two steps in the waveform design procedure. Following works would investigate some improvements of the proposed algorithm for linear programming. It makes the proposed scheme more simple and efficient.

## Figures and Tables

**Table 1:** Simulation Parameters.

Simulation Parameters		
$E_{f}$	Signal power	1
L	Length of transmission waveform	60
SNR	Received SNR	15 dB
λ	Temporal correlation	0.1 s
T	PRI	1 ms
$p_{fa}$	False alarm probability	0.01
